# Social representations of alcohol use among women who drank while pregnant

**DOI:** 10.1186/1940-0640-10-S1-A26

**Published:** 2015-02-20

**Authors:** Jane F Kelly, Catherine L Ward

**Affiliations:** 1 Department of Psychology, University of Cape Town, Cape Town, South Africa

## 

A substantial number of women consume alcohol while pregnant, thereby putting their unborn fetus at risk for developing a fetal alcohol spectrum disorder (FASD) [1]. Despite the fact that some of the highest rates of FASDs in the world have been reported in the Western Cape of South Africa [2,3], little research looks at the experiences of pregnant women who drink and what influences their alcohol use. Gaining insight into the social and psychological processes that contribute to risky drinking during pregnancy will help in guiding interventions that aim to prevent prenatal alcohol use, thereby preventing the occurrence of FASDs [4,5].

Using a qualitative approach, 15 semistructured interviews were conducted in a Western Cape community with women who consumed alcohol during their pregnancy, and two focus group discussions with community members. Data collection aimed to elicit how these women and members of their community construct and make sense of alcohol use [6]. The interview and focus group data were analyzed using thematic decomposition analysis [7].

Alcohol use was consistently represented as a social activity that was heavily influenced by peers. Implicit in this construction was the notion that heavy drinking is a norm within this particular community. Although prenatal alcohol use was stigmatized, it was also understood by the pregnant women and community members as a way of dealing and coping with difficult domestic problems, such as infidelity. For some pregnant women, these problems coupled with the social nature of drinking contributed to their alcohol use throughout their pregnancy. For other pregnant women, access to social support and the desire to have a healthy baby and be a responsible mother contributed to a decision to stop drinking.

Future interventions should take the social context of drinking into account, and rather than ignoring it—as most interventions do—use it to not only shift the social norms that surround heavy alcohol use, but also to support pregnant women to stop drinking. Prevention and intervention initiatives should also take a nonjudgemental and supportive approach that focuses on capitalizing on the moment of pregnancy and on teaching psychosocial skills that enable pregnant women to manage their problems effectively.
